# Characterization of SARS-CoV-2 Mutational Signatures from 1.5+ Million Raw Sequencing Samples

**DOI:** 10.3390/v15010007

**Published:** 2022-12-20

**Authors:** Andrea Aroldi, Fabrizio Angaroni, Deborah D’Aliberti, Silvia Spinelli, Ilaria Crespiatico, Valentina Crippa, Rocco Piazza, Alex Graudenzi, Daniele Ramazzotti

**Affiliations:** 1 Hematology and Clinical Research Unit, San Gerardo Hospital, Via G. B. Pergolesi 33, 20900 Monza, Italy; 2 Department of Medicine and Surgery, Università degli Studi di Milano-Bicocca, Via Cadore 48, 20900 Monza, Italy; 3 Department of Informatics, Systems and Communication, Università degli Studi di Milano-Bicocca, Viale Sarca 336, 20100 Milano, Italy; 4 Computational Biology Research Centre, Human Technopole, Viale Rita Levi Montalcini 1, 20157 Milano, Italy; 5 Bicocca Bioinformatics, Biostatistics and Bioimaging Center—B4, Via Follereau 3, 20854 Vedano al Lambro, Italy

**Keywords:** SARS-CoV-2, mutational signatures, APOBEC, variants

## Abstract

We present a large-scale analysis of severe acute respiratory syndrome coronavirus 2 (SARS-CoV-2) substitutions, considering 1,585,456 high-quality raw sequencing samples, aimed at investigating the existence and quantifying the effect of mutational processes causing mutations in SARS-CoV-2 genomes when interacting with the human host. As a result, we confirmed the presence of three well-differentiated mutational processes likely ruled by reactive oxygen species (ROS), apolipoprotein B editing complex (APOBEC), and adenosine deaminase acting on RNA (ADAR). We then evaluated the activity of these mutational processes in different continental groups, showing that some samples from Africa present a significantly higher number of substitutions, most likely due to higher APOBEC activity. We finally analyzed the activity of mutational processes across different SARS-CoV-2 variants, and we found a significantly lower number of mutations attributable to APOBEC activity in samples assigned to the Omicron variant.

## 1. Introduction

Coronaviruses are different types of viruses infecting humans, providing heterogenous respiratory infections, ranging from a mild to severe phenotype [1]. In December 2019, a novel coronavirus, named severe acute respiratory syndrome coronavirus 2 (SARS-CoV-2), was diagnosed in China, being highly infectious and causing unusual viral pneumonia. These characteristics outlined the onset of the coronavirus disease 2019 (COVID-19), which rapidly became pandemic and widespread all over the world [2]. Manifestations of COVID-19 can vary from case to case, showing a severe course of the disease in a subset of patients, which led to an increase of mortality and consistent economic loss as healthcare and welfare systems experienced unprecedented work conditions [3]. Since the beginning, clinicians have been waiting for novel therapeutic strategies from expanded research activities in order to improve patients’ outcome. As a matter of fact, during the first wave of the pandemic, the management of COVID-19 cases was a problematic struggle until the spread of vaccinations worldwide, which helped as a public health approach to mitigate SARS-CoV-2 transmission and related mortality [4].

Unfortunately, the diffusion of vaccinations against SARS-CoV-2 has not been uniform, since predictable socio-economic aspects are still providing consistent disadvantages for low-income countries to receive vaccines [5,6]. In low-income African countries, the vaccination rate is still under 20%, far away from the percentage achieved by high-income countries [6,7]. The low vaccination rate is known to be associated with higher levels of virus transmission, which, in turn, increases the probability of mutagenesis after every replication process and the subsequent onset of multiple variants [8]. Indeed, SARS-CoV-2 variants have become important to consider as mutations on spike protein domains may occur, thus potentially quenching the vast majority of SARS-CoV-2 vaccines that use spike protein as the main immunogenic target [4,6,8].

The Omicron B.1.1.529 variant became the dominant SARS-CoV-2 strain in December 2021, first detected in South African regions and subsequently spreading worldwide in a few weeks, due to its high transmissibility and capability to infect previously infected or vaccinated people [9]. The mutations characterizing Omicron are able to provide a tighter binding of the spike protein to its ligand angiotensin converting enzyme 2 (ACE2), in addition to a substantial reduction in terms of neutralization activity of both natural and vaccine-induced immunity, thus explaining the selective predominance of this viral strain over the Delta variant (B.1.617.2) and the probability of re-infection in patients exposed to previous variants [9,10].

The low vaccination rate in Africa turned out to be a risk factor in terms of increase of mortality and selection of variants, which further compromised the efficacy of the vaccination itself [10]. Contrary to all expectations, the mortality rate before vaccines and after the start of the campaign remained low, which could be only partially explained by the younger age of African people, suggesting that other genetic and phenotypic factors might play a role in reducing morbidity and mortality in this ethnical group with a low vaccination rate [8,11].

In general, one inborn mechanism involved in contrasting viruses in humans is based on the presence of defense enzymes able to recognize and neutralize exogenous nucleic acids such as virus DNA and RNA [12]. The apolipoprotein B mRNA-editing enzyme catalytic polypeptide-like (APOBEC) family defines a subtype of enzymes able to catalyze cytosine to uracil (C>T) deamination of foreign single-strand DNA, thus providing virus inactivation through genomic mutation [13]. For instance, preclinical studies showed that the APOBEC3 subfamily (A3D/F/G/H) strongly inhibited and inactivated human immunodeficiency virus type 1 (HIV-1) in the absence of the viral protein virion infectivity factor (Vif), which is required by HIV-1 to evade the APOBEC3-related innate immune defense, based on Vif-mediated ubiquitylation and proteasomal degradation of the APOBEC3 complex [14]. Despite this inhibition, a sublethal level of APOBEC3 deamination on HIV-1 complementary DNA (cDNA) is still present, suggesting that Vif and APOBEC3 activities are balanced [12,14]. Interestingly, the disruption of this balance has been studied to further investigate new therapeutic approaches for antiviral therapy, based on the accumulation of deadly mutations preventing HIV-1 replication in host tissues [15]. Furthermore, viral restriction made by APOBEC3 may vary according to the genic polymorphism of this deaminase subfamily and this might have played a role in shaping the HIV-1 epidemic in the African continent [16].

Similarly, some other studies described a C>T transition in the SARS-CoV-2 virus genome as a result of APOBEC3 activity of restriction of viruses and mobile genomic elements, which is further supported by the demonstration of APOBEC3 upregulation in samples derived from hospitalized patients affected by COVID-19 [17]. To date, few data are available that pertains to the collection of samples stating the effective activity of APOBEC3 on the SARS-CoV-2 genome in terms of C>T signature and its relationship with genotypic and phenotypic factors such as ethnicity and clinical outcome [18,19].

## 2. Materials and Methods

In order to provide further insights on this topic, we collected and analyzed 1,585,456 high-quality raw sequencing samples from patients diagnosed with COVID-19 worldwide from January 2020 to April 2022. In detail, we performed a variant calling to obtain a list of mutations for each sample, including both fixed mutations (i.e., with variant frequency (VF) greater than 50%) and minor mutations (with VF less or equal to 50%) [20,21]. We refer to Supplementary File S1 for the list of considered samples.

### 2.1. Variant Calling 

Variant calling was performed by employing the iVar (https://github.com/andersen-lab/ivar (accessed on 6 October 2022), version 1.3.1) recommended pipeline to analyze SARS-CoV-2 ARTIC v3 amplicon sequencing data. We performed the following steps: (1) FASTQ files were mapped to the reference genome SARS-CoV-2-ANC with bwa mem (https://bio-bwa.sourceforge.net (accessed on 6 October 2022), version 0.7.17-r1188). (2) Sorted BAM files were generated from bwa mem results using SAMtools (https://www.htslib.org (accessed on 6 October 2022), version 1.10). (3) ARTICv3 primer sequences were trimmed using the ivar trim command. (4) Trimmed sorted BAM files were built and indexed with SAMtools. (5) Mutation calling was performed from trimmed sorted BAM files using ivar variants. (6) Finally, SAMtools depth was used to extract coverage information from trimmed sorted BAM files.

Quality control was performed on the mutations obtained with ivar variants. We first selected (ultra) deep sequencing samples with a coverage of at least 100 reads in at least 90% of the viral genome. Then, we performed further filtering by selecting only mutations with a variant frequency of at least 5%, where mutations were supported by at least 10 reads, and with a *p*-value resulting from the ivar variants algorithm less than 0.01. Finally, samples with more than 100 minor mutations (after filtering) were removed.

### 2.2. Dataset

We analyzed a dataset obtained from 251 distinct NCBI BioProjects which included 1,585,456 samples (see Supplementary File S1 for the full list). For all samples, only Illumina AMPLICON paired-end high-coverage sequencing data were considered; samples were collected from multiple locations around the world. Within this dataset, we considered for our analyses 862,385 high-quality samples having a coverage ≥ 100 in at least 90% of the virus genome, collected between January 2020 and April 2022.

### 2.3. Mutational Signatures Analysis

Mutational signatures analysis was performed with non-negative matrix factorization and standard metrics were used to determine the optimal number of signatures (rank) as proposed by Maspero and collegues [22].

### 2.4. Pango Analysis

We created consensus sequences as the input to Pangolin [23] from the mutations obtained from raw sequencing data. We considered mutations with a variant frequency > 0.50, i.e., the threshold used for standard consensus sequences. We created consensus sequences for each sample by adding to the reference genome SARS-CoV-2-ANC [21] sequence, the substitutions, insertions, and deletions observed in the sample for each position, and by choosing the one at a higher variant frequency if multiple mutations were detected in the same position. On such inputs, Pangolin version v4.1.2 was executed with default parameters.

## 3. Results

To investigate the existence and quantify the effect of mutational processes causing mutations in SARS-CoV-2 genomes when interacting with the human host, we analyzed the distribution of nucleotide substitutions in our dataset. Fixed mutations are typically transmitted during infections; therefore, they are not representative of mutational processes occurring within a single host [21]. For this reason, we focused on nucleotide substitutions of minor mutations, as proposed elsewhere [20]. We split our cohort in three groups based on the number of minor substitutions observed in a sample: (i) low mutational activity (347,323 samples, 49% of our dataset, where very low or no mutational processes were observed) with samples showing between 1 to 2 minor substitutions; (ii) medium mutational activity (297,168 samples, 42% of our dataset) with samples showing between 3 to 9 minor substitutions; (iii) high mutational activity (65,162 samples, 9% of our dataset) with samples showing at least 10 minor substitutions.

On the one hand, this analysis highlighted that more than half of the considered SARS-CoV-2 samples had very few minor mutations, suggesting very low or absent activity of mutational processes in these patients. On the other hand, the small set of samples (9% of the samples in the dataset) within the high mutational activity group was observed to account for more than 41% of all the observed minor mutations in the dataset.

It has been shown that different mutational processes may generate characteristic mutational patterns in terms of nucleotide substitutions named mutational signatures, which can be computationally extracted from raw sequencing data [20,22]. Mutational signatures computational analyses fall mostly within two categories: (i) de novo discovery of mutational signatures [22,24] and (ii) signatures assignment [22,24,25]. In the first case, the presence of mutational processes is first assessed from the data, signatures are identified and extracted, and finally assigned to samples. Instead, the estimation of signatures assignments is performed by holding a set of signatures fixed and assigning them to samples by minimizing, e.g., the mean squared error between the observed and estimated mutational patterns for each sample.

Accordingly, we: first performed de novo discovery considering only the high mutational activity group in order to guarantee efficient signatures inference [25] and then, given the signatures discovered in the previous step, we performed signatures assignment for both the medium mutational activity group and the high mutational activity group (see Materials and Methods). This approach allowed us to both detect the presence of significant mutational processes causing mutations and to quantify the extent of the activity of such processes. We did not consider the low mutational activity group for this analysis, as mutational processes appear not to be significantly active in such a group.

**Figure 1:**
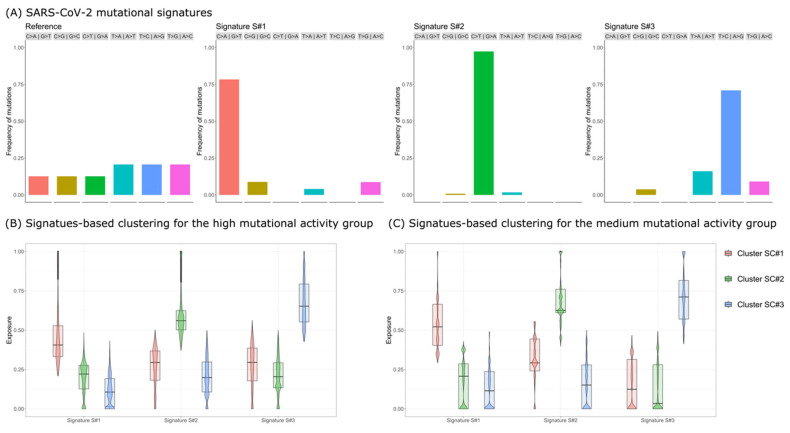
SARS-CoV-2 Mutational Signatures Analysis. (**A**) Mutational signatures of SARS-CoV-2. The barplots return the nucleotide substitution distribution for the reference genome (left) and for the three mutational signatures (S#1, S#2, and S#3) identified via de novo discovery performed on the high mutational activity group (harboring >10 minor mutations; see the main text for further details). (**B**) Signature-based clustering for the high mutational activity group. Clustering is performed on the signature exposure matrix after assigning signatures S#1, S#2, and S#3 to samples (see the Supplementary Material for further details). The boxplots display the distribution of the signature exposure for all clusters and all signatures. (**C**) Signature-based clustering for the medium mutational activity group (>2 and <10 minor mutations).

As a result, we identified three well-differentiated mutational signatures (Figure 1A):

- Signature S#1: mostly characterized by C>A|G>T mutations and previously associated with reactive oxygen species (ROS) activity [20];
- Signature S#2: mostly characterized by C>T|G>A mutations and previously associated with APOBEC activity [19];
- Signature S#3: mostly characterized by T>C|A>G mutations and previously associated with adenosine deaminase acting on RNA (ADAR) activity [20].

Given the three discovered signatures, we then performed signatures-based clustering [20,22,24] for the high- and medium-mutational activity groups and obtained in both cases three well-differentiated clusters, mainly characterized by either one of the three signatures, suggesting that in these samples, mutations were typically caused by either one of the three mutational processes (Figure 1B,C).

**Figure 2:**
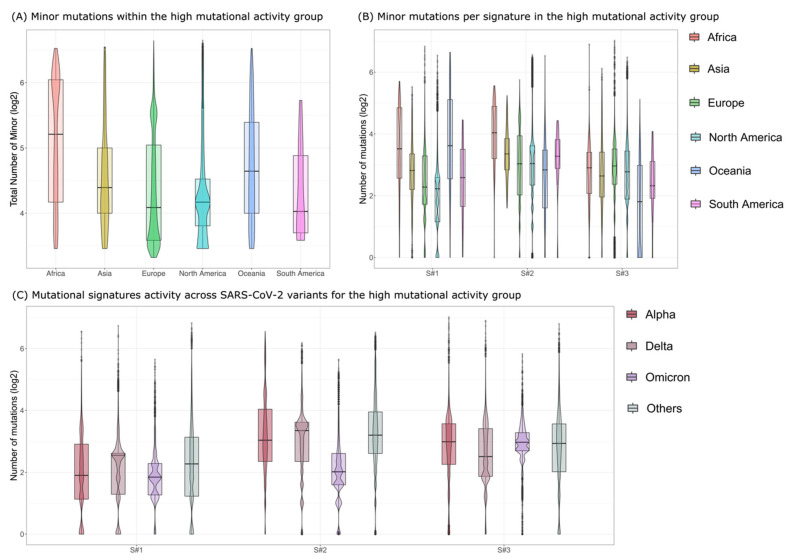
Minor mutations in the high mutational activity group. (**A**) The boxplots show the distribution of the number of minor mutations across the different continents for the high mutational activity group. (**B**) Boxplots showing the number of minor mutations across the different continents grouped by signature for the high mutational activity group. (**C**) Boxplots showing the activity of the three mutational signatures across SARS-CoV-2 variants for the high mutational activity group.

We next assessed the activity of the three mutational processes in different continental groups, by grouping the samples by continent. While no differences were observed in the medium mutational activity group (see Supplementary Figures S1 and S2), the samples from Africa within the high mutational activity group showed a significantly higher number of minor mutations (Figure 2A, standard *t*-test *p*-value = 4.055 × 10^−59^) mostly due to the higher activity of signature S#2 (Figure 2B, standard *t*-test *p*-value = 8.085 × 10^−76^), possibly suggesting higher occurrence of APOBEC-mediated mutations in such samples. Furthermore, we verified the absence of significant sampling bias, which might have explained the observed differences and found no significant impact, which was also due to the very large sample size and long timeline of the analyzed dataset.

We finally analyzed the activity of the three mutational signatures among different SARS-CoV-2 variants. To this end, we first categorized each sample via Pangolin [23] (see Materials and Methods) by considering four major groups: (1) Alpha variant (B.1.1.7 and Q Pango [26] lineages), (2) Delta variant (B.1.617.2 and AY Pango lineages), (3) Omicron variant (B.1.1.529 and BA Pango lineages), and (4) Other, including all the remaining Pango lineages. We report the results in Figure 2C for the high mutational activity group and in Supplementary Figure S3 for the medium mutational activity group.

Interestingly, we found a significant lower activity of mutational signatures S#2 (APOBEC) in the high mutational activity group for the samples assigned to the Omicron variant (Figure 2C, standard *t*-test *p*-value = 0). This result, although intriguing, requires further investigation.

Finally, we performed dN/dS analysis [20] for the three signatures-based clusters within the high mutational activity group (see Supplementary Figure S4) to investigate the presence of selection. While mutations associated with ADAR activity present a pattern of neutrality, ROS- and APOBEC-associated mutations appear to be purified. This is particularly expected for APOBEC mutations.

## 4. Discussion

In this work, we have presented the largest quantitative analysis of minor mutations and mutational signatures of SARS-CoV-2 to date, which allowed us to characterize the mutational processes that are actively causing new mutations in the viral genomes, as well as their prevalence across different geographical locations and virus variants.

We identified three mutational processes, respectively, associated to reactive oxygen species (ROS), apolipoprotein B editing complex (APOBEC), and adenosine deaminase acting on RNA (ADAR), and evaluated the activity of these mutational processes in different continental groups, showing that some samples from Africa present a significantly higher number of substitutions, most likely due to higher editing activity.

Finally, we analyzed the activity of mutational processes across different SARS-CoV-2 variants and found a significantly lower number of mutations attributable to APOBEC in samples assigned to the Omicron variant. We leave to future works the investigation of possible mechanisms leading to such observations.

## Data Availability

All data presented in the manuscript can be downloaded from the original SARS-CoV-2 sequence repositories.

## Supplementary Materials

The following supporting information can be downloaded at: https://www.mdpi.com/article/10.3390/v15010007/s1, Figure S1: Number of minor mutations for the medium mutational activity group, Figure S2: Number of minor mutations per signature for the medium mutational activity group, Figure S3: Signatures activity across SARS-CoV-2 variants for the medium mutational activity group, Figure S4: dN/dS analysis; File S1: Samples information.
