# Supplementary material for: Characterization of SARS-CoV-2 Mutational Signatures from 1.5+ Million Raw Sequencing Samples
Source: Viruses. 2022 Dec 20;15(1):7. doi: 10.3390/v15010007 (PMC9864147; doi:10.3390/v15010007)
Supplement: Supplementary file 1 [file viruses-15-00007-s001.zip › SupplementaryFiguresCaptions.pdf]

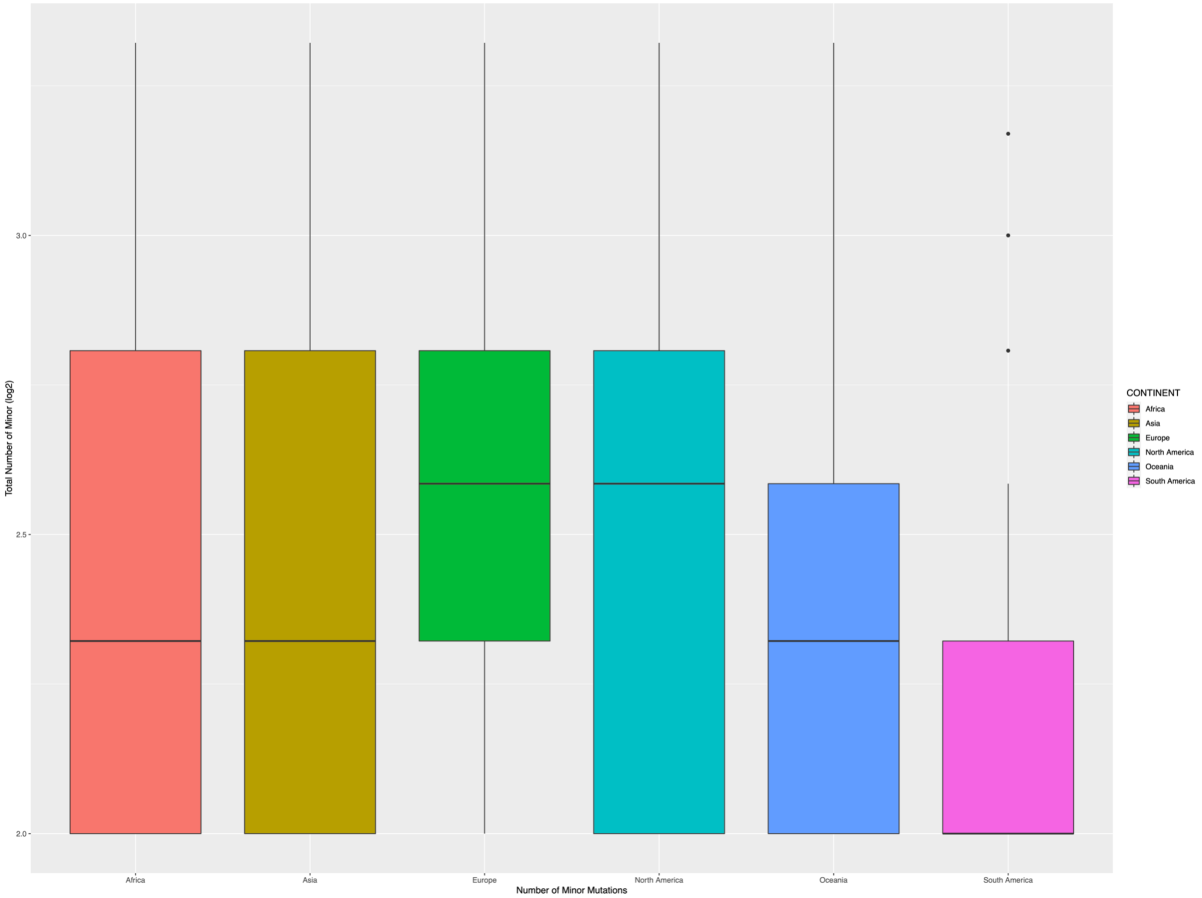

Figure S1. Number of minor mutations for the medium mutational activity group. Boxplots showing the number of minor mutations across the different continents for the medium mutational activity group.

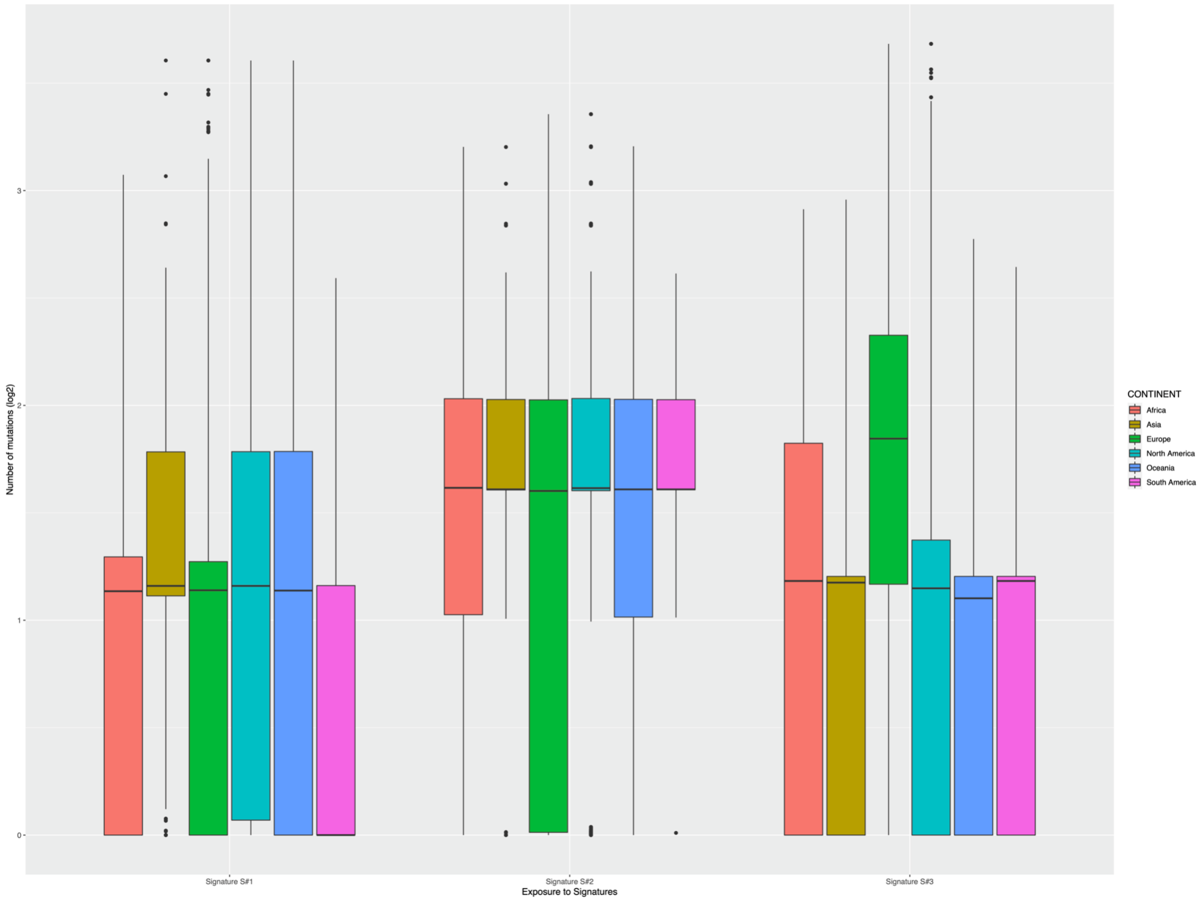

Figure S2. Number of minor mutations per signature for the medium mutational activity group. Boxplots showing the number of minor mutations across the different continents divided per signature for the medium mutational activity group.

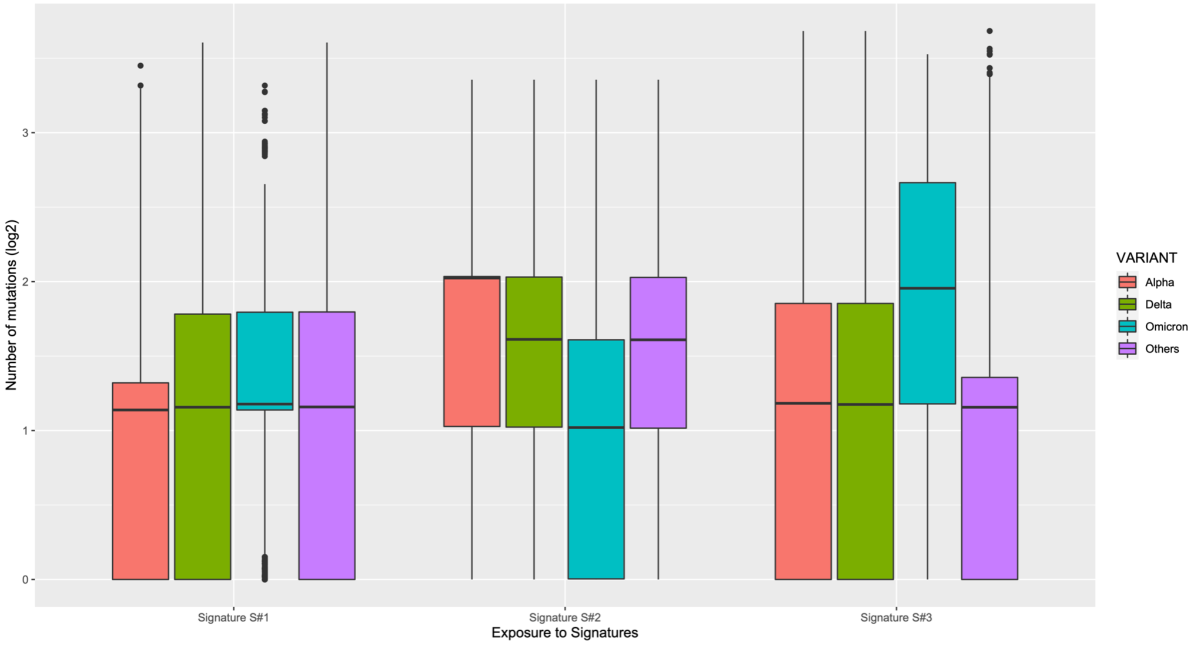

Figure S3. Signatures activity across SARS-CoV-2 variants for the medium mutational activity group. Boxplots showing the activity of the three mutational signatures across SARS-CoV-2 variants for the medium mutational activity group.

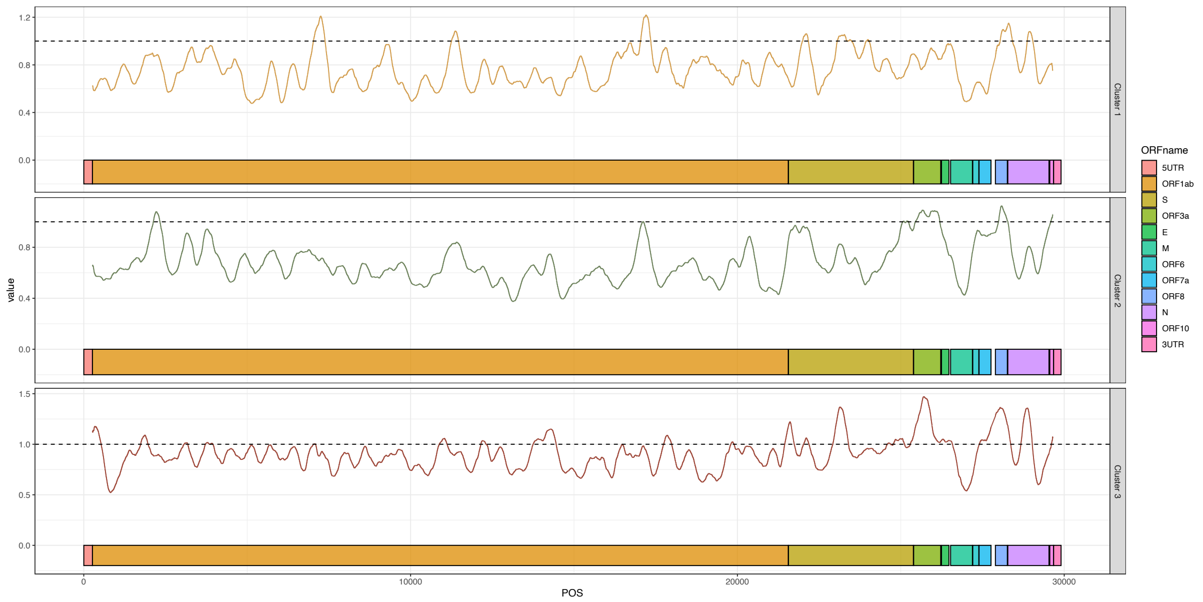

Figure S4. dN/dS analysis. dN/dS analysis for the three signature-based clusters.
